# Design-Time Reliability Prediction Model for Component-Based Software Systems

**DOI:** 10.3390/s22072812

**Published:** 2022-04-06

**Authors:** Awad Ali, Mohammed Bakri Bashir, Alzubair Hassan, Rafik Hamza, Samar M. Alqhtani, Tawfeeg Mohmmed Tawfeeg, Adil Yousif

**Affiliations:** 1 Department of Computer Science, College of Science and Arts—Sharourah, Najran University, Sharourah 68341, Saudi Arabia; aaomar@nu.edu.sa; 2 Department of Math, Turubah University College, Taif University, Taif 26571, Saudi Arabia; safi@tu.edu.sa; 3 Department of Computer Science, Faculty of Computer Science and Information Technology, Shendi University, Shendi 41601, Sudan; 4 Department of Computer Science, School of Computer Science and Informatics, University College Dublin, Belfield, D04 V1W8 Dublin, Ireland; alzubair.mohamedtahir@ucd.ie; 5 Lero-the Irish Software Research Centre, Tierney Building, University of Limerick, Sreelane, V94 NYD3 Limerick, Ireland; 6 Big Data Integration Research Center, National Institute of Information and Communications Technology, Tokyo 184-8795, Japan; rafik.hamza@nict.go.jp; 7 Department of Information Systems, College of Computer Science and Information Systems, Najran University, Najran 61441, Saudi Arabia; smalqhtani@nu.edu.sa; 8 Department of Computer Science, Faculty of Computer Science and Information Technology, University of Science and Technology, Khartoum 14411, Sudan; tawfeeg.mohammed@ust.edu.sd

**Keywords:** software design, architecture-based prediction, component-based, reliability, software quality, sensors

## Abstract

Software reliability is prioritised as the most critical quality attribute. Reliability prediction models participate in the prevention of software failures which can cause vital events and disastrous consequences in safety-critical applications or even in businesses. Predicting reliability during design allows software developers to avoid potential design problems, which can otherwise result in reconstructing an entire system when discovered at later stages of the software development life-cycle. Several reliability models have been built to predict reliability during software development. However, several issues still exist in these models. Current models suffer from a scalability issue referred to as the modeling of large systems. The scalability solutions usually come at a high computational cost, requiring solutions. Secondly, consideration of the nature of concurrent applications in reliability prediction is another issue. We propose a reliability prediction model that enhances scalability by introducing a system-level scenario synthesis mechanism that mitigates complexity. Additionally, the proposed model supports modeling of the nature of concurrent applications through adaption of formal statistical distribution toward scenario combination. The proposed model was evaluated using sensors-based case studies. The experimental results show the effectiveness of the proposed model from the view of computational cost reduction compared to similar models. This reduction is the main parameter for scalability enhancement. In addition, the presented work can enable system developers to know up to which load their system will be reliable via observation of the reliability value in several running scenarios.

## 1. Introduction

As reliance on software applications is growing, software reliability analysis is unavoidable. Software reliability is defined as the probability that reveals the capability of a system to perform a required service or function correctly (failure-free) in a certain environment, over a particular time [1,2]. A failure is an incorrect result or unexpected behavior impacting the requirements of the software. Reliability prediction models participate in the prevention of software failures which can cause critical events and disastrous consequences in safety-critical applications or even in businesses.

Depending on the reliability measurement lifecycle, the measures can be taken early in development, or later in the testing or post-deployment [3,4,5]. The data used in the development are appraisal data, whereas testing data and real-world data are used for the later measurements. The early measurements are performed to discover weak design spots. On the other hand, the later measurements are used for component certifications and system release decisions. The reliability models used for later measurements (testing and post-deployment) are similar because there is no big difference between input data and the objectives. For instance, the early reliability models focus on tackling the problems related to the shortage of operational data before the coding and the way of modeling system behavior precisely. The later models mainly focus on the accuracy of the prediction model without much consideration of the approaches of data elicitation and modeling of system behavior [6,7]. This research is oriented toward early reliability measurement because it is the most cost-effective regarding time and budget, by avoiding reconstructing the entire system or repeating testing.

The main goal of design-time reliability models is to predict the reliability of software based on information related to the software architecture made out of components. Key potential benefits that may be obtained from using architectural design artifacts as input for the reliability model can enable comparing of different architecture options or design alternatives. In addition, studying the sensitivity of complete application reliability to the reliabilities of components and identifying critical components and scenarios. Furthermore, this kind of model assists with a better understanding of the reliability requirements and how they relate to the architectural design.

Software behavior models that are derived from requirement specifications [8,9,10,11,12] are basic building blocks for design-time reliability predictions. Many studies have been carried out based on these behavior models to predict the reliability at early design stages [7,13,14,15,16,17,18]. However, the current models need to pay more attention to scenario combination mechanism and model scalability for larger systems [8,9,10,19]. The scalability problem of the model is related to system behavior modeling. The behavior modeling is concerned with moving from scenario specifications to state machine specifications. Unlike most reliability models, our proposed model moves from system specification explicitly using a scenarios description language. Developing a scalable behavior modeling approach is a key for enhancing the scalability of the reliability model. The behavior approach can provide suitable scenario modeling that considers the size of each scenario and the number of scenarios. The solution starts by describing the system’s scenarios in a compact and concise manner, which leads to fewer scenarios. At the same time, the reliability model needs to provide solutions that can mitigate computational complexity which is another factor that hinders the scalability, by introducing a suitable reliability calculation strategy. The main factors that can lead to complexity mitigation are (1) the reduction in number of state machines obtained from the system behavior model and (2) the reduction in the states within the state machine itself. In the current models these two factors are tackled partially. Some of the models reduce the internal states of the individual state machines and neglect the number of state machines, and the other models reduce the state machines without consideration of the number of the internal states of the state machine itself.

Most reliability models that lack scalability rely directly on Markov notation (state machine specifications) as their primary modeling notation and give no details on how the states are derived. Moving to Markov provides a mathematical formula to calculate reliability. However, this can lead to a state-space explosion problem [20]. In the proposed model, the calculation of reliability is conducted partially. Given the scenarios represented in the form of states, the calculation implemented via mathematical formula partially deals with the state space. This way of partial calculation enables the computations to be carried out with limited state space, which can mitigate the computational complexity [19,20].

The first part of this paper shows a strategy for scalable scenario modeling and reliability calculations. It adapts our previous scenario description language named scalable triggered scenario (*s-TS*), which was introduced in [12]. The *s-TS*s helps in describing scenarios of a system compactly and concisely, leading to fewer scenarios. The *s-TS* in our previous work [12] was used to obtain individual components’ behavior models, whereas in this paper, it is utilized for analysis of the complete behavior of the scenarios to predict the reliability of the scenarios and then the whole system. The first part of this paper also includes an approach toward improving scenarios’ traceability by avoiding computational complexity. System scenario in the approach is translated to finite state machines (FSMs): each FSM illustrates the behavior of a component instance inside the scenario. The failure probability of each component that appeared in the scenario is separately calculated depending on the FSM. Finally, a modified mathematical formula is used to calculate the whole scenario’s reliability based on the components’ behavior models and their related failure information.

In the second part of this paper, the scenarios’ reliabilities obtained from the first part are used as inputs to calculate system reliability. Firstly, the system reliability is calculated for sequential software applications. The ATM case study illustrates the calculation as an example of sequential applications. Secondly, the method for predicting the concurrent software applications’ reliability is introduced. A multinomial statistical distribution [21] is used in this method as the main part of a scenario combination process. Furthermore, a binomial distribution as another probability distribution is used to generate the probabilities of scenarios’ failure. Finally, the automated railcar system case study [22,23,24] as a concurrent software application is used to illustrate the prediction method. The automated railcar is a sensor-based application consisting of four components—namely, a car, a cruiser, a carHandler, and a proxSensor. More details related to the views and scenarios selected for the case study are provided in Section 3 and Appendix B.

In the final phase of the study, the performance of the proposed model is evaluated by comparing its performance with current models. The evaluation explores the computational cost reduction regarding the space needed to deal with the system’s scenarios. Compared to the existing works, the proposed model demonstrated more efficient performance.

## 2. Related Work

In the context of design-time reliability prediction, various models [7,13,19,25,26,27,28,29,30,31,32,33] have been developed for the early design stages. These models can be classified into gray-box [30,31,32,33] and white-box [7,13,19,25,26,27,28]. In the gray-box models, system reliability is predicted based on the components’ reliability without knowledge of the components’ internal behavior. The components’ reliability is estimated using one of the existing black-box techniques. In contrast, the white-box models build their techniques for estimating individual components’ reliability based on their architectural design, which describes their internal behavior. Our proposed model contributes and supports architecture-based white-box modes. Furthermore, rather than relying on components reliabilities, the proposed model goes into more depth on components’ internal behavior. The individual components’ operations that participate in the interactions within the scenario are taken into account to obtain the reliability of the scenario. The term scalability refers to modeling large and complex software systems. The software system’s size and its complexity depend on the number of system operations [9,34,35] and scenarios rather than the number of components [8,9]. The system’s complexity is measured by counting the number of operations within the individual scenarios [8]. Computational cost incrementation is always the result of these two factors. To compute the reliability of a complex scenario, there is a need for enough space to fit the operations and the transitions among these operations. The number of scenarios affects the computational cost because there is also a need for large space to solve the final model built through scenario combination.

The scalability problem is one of the main issues that have been neglected by the current reliability models [20], except for the study presented by Cheung et al. [36]. The increase in the computational cost is a problem associated with scalability. Solving the scalability problem often leads to large state space, resulting in increased computational cost. In the approach presented in [36], scalability and computational cost reduction were considered. It tackles scalability by truncating the scenario to sub-scenarios based on a hierarchical organization. Meanwhile, the separate computation of reliability of each sub-scenario results in a reduction in computational cost. The study presented in [33] introduced a solution for computational cost reduction that depends on state-space compression. However, the method in [33], unlike [36,37] and our proposed method, does not depend on the requirement specifications as the main sources for the scenario synthesizing.

Developing a scalable behavior modeling approach can play a central role in enhancing the scalability of the reliability prediction by providing suitable scenario modeling that considers the size of the scenario and the number of scenarios. Exactly, the reliability approach should solve the scalability problem by describing the system’s scenarios in compactly and concisely manner, which leads to fewer scenarios. At the same time, it can create a balance between scalability and computational cost by introducing a suitable reliability calculation strategy. Software reliability models that are designed for traditional programming techniques, such as procedural and object-oriented programming, are incapable of analyzing the reliability of the modern software methodologies. The current software, such as CBS and SOA, requires new reliability analysis approaches to differentiate between sequential and concurrent applications. In the sequential application, the execution transitions move from one scenario to another in a sequence, whereas in the concurrent application, the execution transitions move to multiple scenarios running concurrently. Therefore, predicting the reliability in sequential software is different compared to concurrent software [15]. In recent literature, most approaches have not paid much attention to the nature of the concurrent applications and their required modeling [16,33].

Table 1 summarizes our findings regarding the features supported by our proposed model compared to current design-time software reliability prediction models. These features are (1) representation of the nature of the concurrency applications, (2) components’ behavior tackled at operations level and (3) scalability enhancement. A checkmark ✓ indicates supporting the feature fully, and the cross × indicates not supporting it. A checkmark in parentheses (✓) means that a model partially supports the feature. The models presented in Table 1 can be classified into three groups. The first group includes the models in [7,25,28]. These models use behavior modeling tools built on architecture descriptions and UML diagrams, such as use case diagrams (UCD), statechart and component diagrams (CDs). The behavior modeling tools are named rich architecture definition language (RADL), Palladio component model (PCM) and communicating automata with probabilistic transitions (CAWPTs), respectively. These behavior modeling tools produce finite state machines (FSM) or the notations of the Markov Model (MM) as outputs. The reliability is calculated based on discrete-time Markov chains (DTMC) or continuous-time Markov chains (CTMC). However, these tools are not available to download or as commercial products, unlike others. Moreover, the sequence chart, which can control time-dependent behavior system modeling, is not part of any of these models.

The models presented in [16,30,35,36] represent the second group, which uses sequence diagrams (SDs) or message sequence charts (MSCs), which are pretty similar. SDs and MSCs use a link to facilitate the conversion from system specifications to MM. SDs and MSCs are powerful scenario description tools. However, the *s-TS* scenario language presented in our proposed model is similar to SDs and MSCs. Furthermore, it provides more solutions toward scalability enhancement by describing the scenarios compactly and concisely. The third group is represented by the models in [13,19,27,31,33]. In these models, MM notation is used directly to generate probabilistic dependency graphs (PDG) to model the interactions among components and to calculate reliability algorithmically based on PDG. PDG and MM can be mapped to CTMC or DTMC for reliability calculations. Most of the models presented in the table lack support for the three features checked in the columns in Table 1, though there are exceptions [16,31,33] that support the features partially. Solutions for scalability enhancement must be provided in behavior modeling and reliability calculations.

Behavior modeling needs tools that can facilitate the move from system specifications to MM. Furthermore, we need scalability and time-dependent behavior modeling capabilities. Modeling tools are preferred to provide UML-like notation familiar to the system developers. For example, solutions that relate to the facilitation of the conversion from system specifications to MM are presented in [7,16]. Such a solution is required for a realistic interpretation of the model by the system’s architect. In addition, more strategies for scenario division and compression, presented in [16,31,33], are required to support scalability.

## 3. The Proposed Model

**Figure 1:**
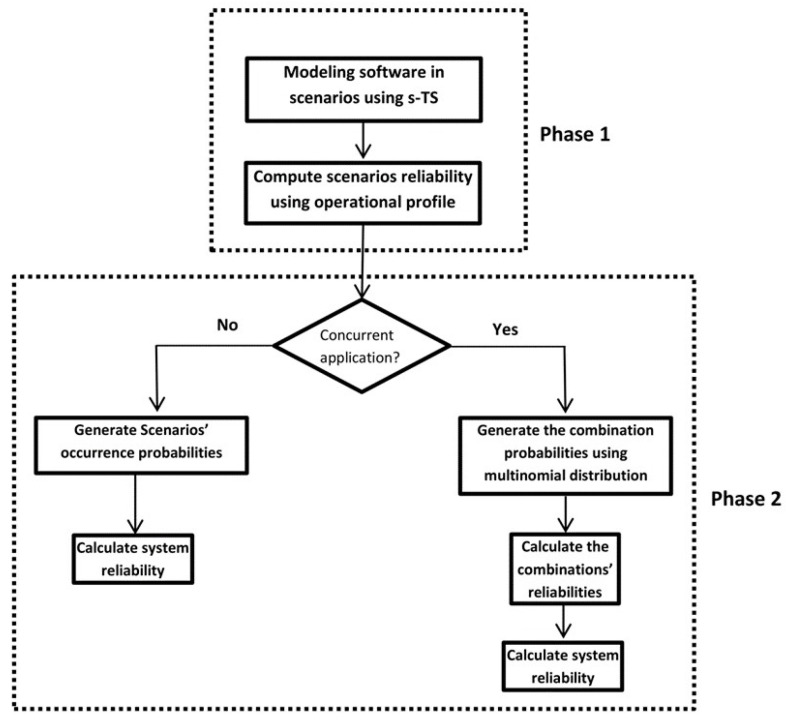
The proposed model’s phases and activities.

The proposed model has several activities, as detailed in Figure 1. These activities are divided into two main phases. The first phase illustrates the activities required for behavior modeling and scenarios’ reliability calculation. The second phase describes the scenarios’ combination mechanism and the determination of system reliability.

As shown in Figure 1, model activities start by describing system scenarios using *s-TS* [12]. The activities of phase one are illustrated using a run-time example in Section 3.1. Briefly, several behavior modeling steps described in our previous work [12] are applied in phase 1. As described in Section 3.1, state machines will construct for all components instances within a scenario. Then, in Section 3.2, the failure probability associated with each component instance is calculated by treating its FSM separately. Hence, the calculation of scenario reliability is conducted in limited state space to reduce the computational cost. The component’s failure probability reveals the criticality of the component regarding the scenario. Then, scenario’s reliability in Section 3.2 is computed using operational profile information. At the end of phase 1, the failure probability of the whole scenario is calculated based on the failure probabilities of the components. The importance of tackling components’ behavior independently helps construct the global system behavior model without the need for components’ internal states (components interactions), which in large software systems leads to the state-space explosion problem. In phase 2 of Figure 1, which is detailed in Section 3.3 of this paper, for sequential applications, the system reliability is calculated based on the table of scenarios’ occurrence probabilities. In concurrent applications (Section 3.3.2), multinominal distribution is used to generate combination probabilities of scenarios toward calculating combination reliabilities. The final results of these activities are system reliability under the assumption of concurrent execution.

### 3.1. Behavior Modeling

Describing system scenarios using *s-TS* as a scenario description language annotated with system constraints can force system architects to know the requirements and the system behavior implied by these scenarios. This is due to *s-TS* language identifying clear causality relations among dissimilar behavior directions via conditional, triggered and preemptive behavioral representations. The most important characteristic of *s-TS* is the enhancement of the scalability by describing software scenarios in a concisely and compactly way, which leads to fewer scenarios. However, the scenario’s compaction can increase the number of states within the scenario, thereby increasing the computational cost. However, this can be avoided by truncating each scenario into small units in the reliability calculation based on the component instances.

#### 3.1.1. Running Examples

**Figure 2:**
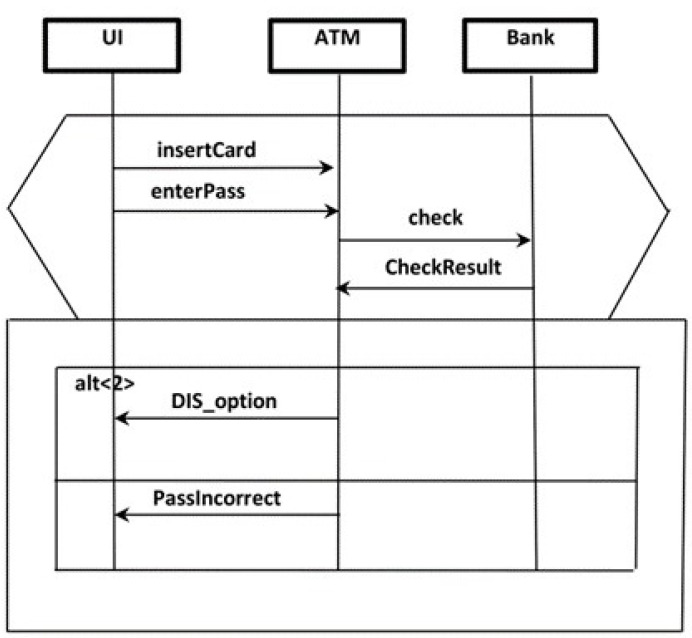
The password verification scenario of the ATM system.

**Figure 3:**
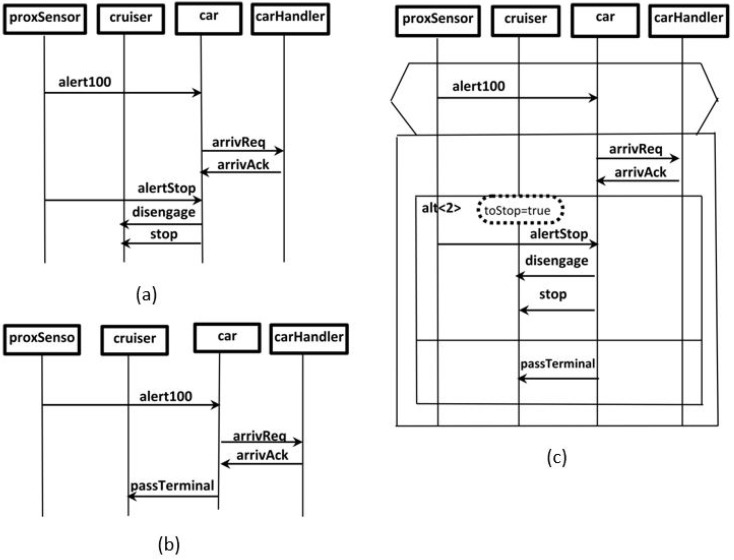
A portion of a railcar system case study is described in two sequence charts, combined by *s-TS* into one chart. (**a**) Simple sequence chart describing scenario of car approaching terminal with stopping at terminal. (**b**) Another simple sequence chart describing scenario of car approaching terminal with passing that terminal. (**c**) One *s-TS* combining the two scenarios described in (**a**,**b**).

The password verification scenario of an ATM (shown in Figure 2) was selected as an ongoing example to illustrate the proposed model. As shown below in Figure 2, the chosen scenario consists of three components named UI, ATM and Bank. In addition, the scenario shown in Figure 3 of the automated railcar system [22,23,24] is also used in this section as a complementary ongoing example to illustrate the behavior modeling. However, in Section 3.2, the reset scenarios of the ATM will depict the calculations of scenario reliability. Furthermore, in Section 3.3, which illustrates the measure of system reliability, the ATM system is used to represent sequential software applications, and the railcar system is used for concurrent applications. For more clarification, Appendix A and Appendix B. contain the scenarios’ specifications and the related documents of the two case studies.

#### 3.1.2. Scenario Preparation

The scenario preparation starts by modeling the scenario using *s-TS* [12]. *s-TS* is similar to most existing triggered scenario description languages [8,9,10,38]. It was derived from the basic syntax of sequence charts named labeled partial orders (LPO). *s-TS* uses additional constructs to make scenarios more compact and concise. Therefore, descriptions of system scenarios in any sequence chart syntax can be considered input for *s-TS* scenarios. *s-TS* is a tool that can describe or combine scenarios compactly and concisely. Figure 3c shows a *s-TS* scenario which is a result of combining scenarios shown in Figure 3a,b that were described in sequence charts. A scenario in *s-TS* is a triple (T,M,b−msg), where *T* is a trigger chart; *M* is a main chart; and b−msg is called the branching message, which can be located in *T* or *M*. The trigger chart holds the events that trigger the scenario, and is represented by LPO with ∑ (event alphabet). The *M* chart holds the events that occur in response to the trigger events, and is represented by a set of LPO with ∑. Moreover, the main chart has an alt operator and implies triggers as other held elements.

**Figure 4:**
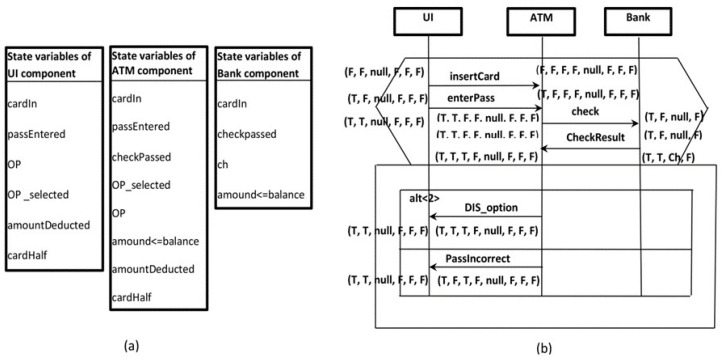
The scenario of password verification after the annotation and propagation. (**a**) State variables of the components participate in the scenario which is the main input for annotation. (**b**) The scenario of password verification that annotated and propagated using the state variables shown in figure (**a**).

The scenario was prepared similarly to that described in our previous work [12], though all the components within the scenario were annotated and propagated simultaneously and not only one component instance, as in [12]. Herein, the targeted behavior model represents the behavior of the whole scenario, whereas in [12] the constructed behavior model represented the behavior of one component. Figure 4b shows the scenario of password verification that has been shown in Figure 4 after the annotation and propagation. Each component instance is annotated independently based on its state variables, as shown in Figure 4a.

**Figure 5:**
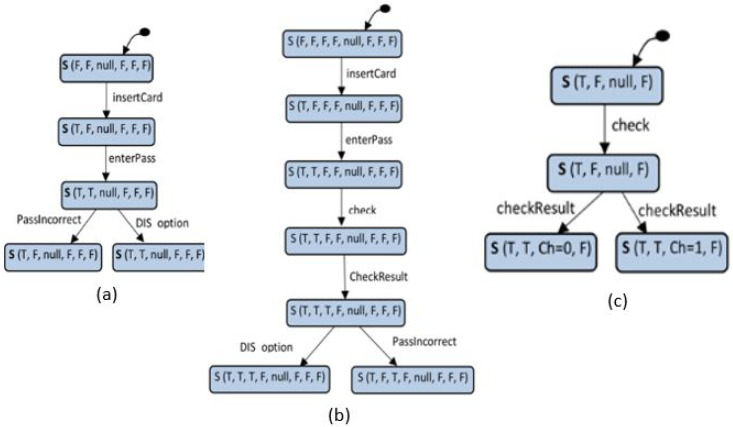
The FSMs obtained from the password verification scenario. (**a**) FSM UI component. (**b**) FSM of ATM component. (**c**) FSM of Bank component.

Translation of the prepared *s-TS* scenario to FSMs. Once the system scenario of *s-TS* Translation of the prepared *s-TS* scenario to FSMs. Once the system scenario of *s-TS* is annotated and propagated, it will be ready to translate to FSMs. These FSMs represent the behavior of the scenario. This procedure translates each component instance depicted in the scenario to FSM. For example, given an *s-TS*, let $F_{s\text{-}TS}^{i}=\{S,S_{0},S_{b},T\}$ be an FSM obtained from instance i in *s-TS* . The idea is to translate each state vector value to a state in the FSM. Therefore, the normal states corresponding to the state vector values within the component instance are represented by *s*. At the same time, *S*_0_ represents the first state vector value. If *S_b_* represents a branching state vector value, send messages and replay messages of the component instance are represented by *T* as a transition relation. Figure 5 shows FSMs of the scenario of password verification translated from the scenario shown in Figure 4b.

### 3.2. Calculating a Scenario’s Reliability

In calculating the scenario’s reliability, each FSM obtained is tackled separately to avoid computational complexity. Therefore, the formulas and discussion of the results obtained via these formulas are presented in the sub-sections below.

The formulas: Assume *k* components $\left(comp_{1},comp_{2},\ldots ,comp_{k}\right)$ belong to scenario *j* $\left(Sc_{j}\right)$. Let the failure probability of $comp_{i}$ be $f_{i}$, which can be a known value. Let the invocation number of $comp_{i}$ within $\left(Sc_{j}\right)$ be $n_{i}$. Based on the work presented by Singh et al. [39], the value of $f_{ij}$, which represents the probability that $comp_{i}$ fails in $\left(Sc_{j}\right)$, can be calculated by Equation (1).

(1) $$f_{ij}=1-{\left(1-f_{i}\right)}^{n_{i}}$$

Based on Equation (1), if we assume that the failure probabilities of the components within each scenario are independent, the failure probability of $Sc_{j}\left(f_{Sc_{j}}\right)$ can be calculated using Equation (2):

(2) $$f_{Sc_{j}}=1-\prod_{i=1}^{K}{\left(1-f_{i}\right)}^{n_{i}}$$

The scenarios described by *s-TS* can participate in discovering inconsistencies related to component interactions. These inconsistencies cause errors such as a signature mismatch of interfaces and pre- and post-conditions. The definition and categorization of the errors caused by these inconsistencies are beyond this paper. More details of such errors can be found in [38]. In the component-based model, the interactions of the components are managed through components’ required and provided services, which are implemented by the components’ operations. Therefore, all types of these errors can be abstracted and encapsulated in the failure probabilities of operations. In our previous work [38] and the work presented by Singh et al. [39], the components’ failure probabilities were combined into one metric. In this paper, a failure of the operation as an instance of component failure is used as input for Equation (2); therefore, our calculation should be expected to be more accurate.

From the scenario specifications depicted by *s-TS*, the operation invoked for each specific component’s interaction can be determined exactly. Therefore, going back to Equation (1), a modification can be made via replacing it by representing the failure probabilities of the invoked operations within the scenario. Thus, Equation (1), after this modification, can be described as follows:

(3) $$f_{ij}=1-\prod_{l=l_{i}}^{l_{n}}\left(1-f_{i}^{l}\right)$$

Based on Equation (3), the information required to calculate $f_{ij}$, which represents the probability that the component *i* will fail in scenario *j*, is the operational failure probabilities. Table 2 below shows information describing operations of the password verification example and their related failure probabilities. As we mentioned in the previous paragraph, these failure probabilities are the values of $f_{i}^{l}$. The number of techniques presented in [40,41] can be used to estimate $f_{i}^{l}$. The techniques use the analysis of dynamic complexity, connector couplings and the severity levels of failures of the components and the related operations. In the case of FSM used in our behavior model, the numbers of nodes and transitions can be used as parameters for complexity analysis. The reliability models that use behavior models consist of the state chart diagrams and timed sequence diagrams. Information describing the length of a component’s busy period is utilized as a parameter to estimate an operation’s failure probability [39].

By recalling Equations (2) and (3), we can assume that the components’ failure probabilities are independent. The failure probability of the scenario $Sc_{j}$ that is already defined in Equation (2) can be redefined in Equation (4) below based on the $f_{ij}$ of Equation (3).

(4) $$f_{ij}=1-\prod_{i=1}^{K}\prod_{l=l_{i}}^{l_{n}}\left(1-f_{i}^{l}\right)$$

Basic assumptions related to Equations (3) and (4) are that the operations’ failures are independent and the scenario flow is sequential. However, one of the essential characteristics of *s-TS* is producing branching scenarios. Therefore, branching scenarios are expected (e.g., see the three FSMs in Figure 5). To be more accurate, in the application of Equations (2) and (4), each branch is tackled as a sequential structure. Then, the average of the branches must be calculated before the subtraction with Equation (1).

The reliability of the scenario *j* is depicted as the probability of there not being a failure; thus, the reliability of scenario *j* can be calculated as follows:

(5) $$R_{Sc_{j}}=1-f_{Sc_{j}}$$

Results of scenarios’ reliability calculations. Based on the FSMs obtained from the *s-TS* scenario specification, where each component instance participates in the scenario represented by an FSM, it is time to calculate the scenario’s reliability. Firstly, the component’s failure probabilities are calculated using the expected failure values of the operations invoked by the components as inputs to Equation (3). Therefore, applying that to the scenario of password verification, the failure probabilities of the components UI, ATM and Bank were: 0.0054900, 0.0203560 and 0.0069880, respectively. These values reveal critical components concerning the scenario and so the system. Without consideration of the components’ branching structure, by applying Equation (4), the failure probability of the password verification scenario is 0.03544306. Using this value of scenario failure probability as input to Equation (5), the reliability of the scenario is 0.9645569. When taking into account the components’ (cruiser and car) branching structure, the failure probability of the scenario is 0.0325424, and therefore, the reliability of the scenario is 0.9674576. The reliability value obtained by considering the branching structure differs from the other value obtained without this consideration by 0.0038140. This difference may appear to be relatively small. The difference is small due to the depth of the branches being great; however, in the case of long branches, the mean is necessary because the difference will be too big, and thus affect the prediction accuracy.

The other scenarios’ reliabilities in the ATM case study were calculated using the same strategy. The reliabilities of scenarios 2 and 3 are 0.9635422 and 0.9468746,, respectively. Moreover, the strategy was applied to a railcar system case study. The case study consisted of three scenarios: a passenger in a terminal (scenario 1), a car departing from a terminal (scenario 2) and a car approaching a terminal (scenario 3). The reliabilities of these scenarios were 0.9411, 0.9284 and 0.952996, respectively. These values obtained from these two case studies are important to illustrate the idea of calculating system reliability based on scenarios’ reliabilities, which is focused on in the rest of this paper. The ATM case study will be used as an example for the sequential software applications and the railcar for the concurrent applications.

### 3.3. System Reliability

The approaches for predicting and analyzing a software system’s overall reliability based on its sub-scenarios depend on the nature of the software application. The software applications can be classified as sequential and concurrent. In sequential applications, the execution transitions move from one scenario to another in a sequence, whereas in concurrent applications, the execution transitions move to multiple scenarios running concurrently. Therefore, predicting the reliability in sequential software cases is different compared to concurrent cases [15].

#### 3.3.1. The Reliability of Component-Based Sequential Applications

As sequential systems allow one scenario to be active, the system’s reliability $\left(R_{sys}\right)$ is calculated as a weighted mean of scenarios’ reliabilities. The weight $\left(w_{j}\right)$ in Equation (6) below denotes the occurrence probability of scenario j. For example, suppose the occurrence probabilities of scenarios 1, 2 and 3 of the ATM example are 1, 0.10 and 0.90, respectively. The reliabilities of these scenarios, as calculated previously, were 0.9645569, 0.9635422, and 0.9468746, respectively. Based on these values and the application of Equation (6), the reliability of the ATM system as a sequential system is 0.9565492.

(6) $$R_{sys}=\frac{\sum_{j=1}^{n}W_{j}R_{Sc_{j}}}{\sum_{j=1}^{n}W_{j}}$$

#### 3.3.2. The Reliability of Component-Based Concurrent Applications

In a concurrent model representing the entire system’s behavior in *n* communicating scenarios, at any point in time, the system’s state is represented by a set of active scenario instances $x_{1},x_{2},\ldots ,x_{n}$, where n is the total number of the active scenario instances in the system, and $x_{j}$ corresponds to the number of active scenario instances corresponding to the *j*th scenario. The distribution of scenario combination probabilities can be identified through a discrete multinomial probability distribution [21], which is designed to represent the joint behavior of frequencies of each of *j* of the possible outcomes arising from n independent trials. Note that in this paragraph and the next paragraph, the same variables that describe the scenarios and their combinations’ probabilities are used purposely in the definitions of the multinomial distribution to facilitate the mapping between them.

The multinomial distribution is the generalization of the binomial distribution that models repeated choices between two categorical outcomes. A multinomial describes more than two dichotomous categorical outcomes. More details about this distribution and its properties can be found in [42,43]. The multinomial distribution was selected because with each category having a given fixed success probability that highly matches the situation of individual scenarios, the probability distribution function of the multinomial (Equation (7)) gives the probability of any particular combination of numbers of successes for the distinct categories. The success probabilities can be mapped to the occurrence probabilities of the individual scenarios. In the reliability that is built based on the scenario’s reliability, system developers need to know to which load the system will be reliable. The load here refers to the number of total active scenario instances in the system that is represented through the variable *n* in multinomial distribution. Moreover, the multinomial distribution allows further analysis, where the reliability can be predicted based on a different adjustment relevant to the type of set scenario instances. The active instances set can be from the same scenario or different scenarios. More details about this analysis are depicted through the example of railcar system in the next paragraphs.

Therefore, the discrete probability function of multinomial distribution defined in Equation (7) can be used to generate probabilities of various combinations of scenario instances in the system.

(7) $$p\left(x_{1},x_{2},\ldots ,x_{j}\right)=\frac{n!}{x_{1}!,x_{2}!,\ldots ,x_{j}!}p_{1}^{x_{1}},p_{2}^{x_{2}},\ldots ,p_{j}^{x_{j}}$$

As defined previously, *n* represents the total number of the active scenario instances and $x_{j}$ the number of active scenario instances corresponding to the probability of each scenario *j*.

The maximum number of active scenario instances in the software application depends on the decisions related to design, limitations of system resources, requirements, the behavior of components, and the system’s structure. Therefore, in the reliability prediction, the total number of active scenario instances in the system may not be realistically assumed to be unbounded [36]. Hence, assigning a specific value to n is required, and this will control the maximum number of active instances possible for each scenario.

As a good example of the concurrent systems, suppose the reliability of the railcar system is required under the assumption that the maximum number of allowed active scenario instances at a time is three. Based on the concurrency nature of the system, these scenarios can be run simultaneously. There are many cars and terminals in a railcar system. The case study consists of three scenarios: a passenger in a terminal (scenario 1), a car departing a terminal (scenario 2) and a car approaching a terminal (scenario 3). Assume the occurrence probabilities of these three scenarios $p_{1},p_{2}$ and $p_{3}$ are 30,50 and 20, respectively. The occurrence probabilities can be determined based on information relevant to passenger arrival rate and the service time. Based on the application of the multinomial distribution function defined in Equation (7) and the utilization of the p values as input, Table 2 provides various combination probabilities for the scenarios in the railcar system. The values in Table 2 were generated under the assumption that the maximum number of allowed active scenario instances in the system is three (n≤3).

The values of Table 3 can be interpreted as follows. Let us to interpret the value *p* $(x_{1}=1,x_{2}=0,x_{3}=2)=0.036$. This value means the probability of having three active scenario instances (n=3) in the system—where one instance belongs to scenario 1, no instances belong to scenario 2 and two instances belong to scenario three—is 0.036. Due to the restriction that the maximum number of the active scenario instances in the system is three, for each $x_{j}$ variable whose values do not satisfy the condition $x_{1}+x_{2}+x_{3}⩽3$, the occurrence probability is zero. In addition, if it is necessary to determine the reliability of running a specific number of instances, for instance, when the system has exactly three scenario instances, only the cases that satisfy the condition $x_{1}+x_{2}+x_{3}=3$ (shown in Table 4) will be considered in the calculation. However, many tables similar to Table 3 can be extracted from Table 3 by changing the *n* assumption.

#### 3.3.3. The Reliability of the Scenario Combination

The reliability of the scenario combination can be calculated based on the definition of the system failure [36]. The definition may differ from one system to another. For instance, in a critical application such as a railcar system, any scenario instance fails directly and leads one to consider that the whole system has failed. In other systems that have less criticality, the system’s failure may be related to the failure of all the active instances of the same scenario. Hence, any running instance of the scenario will be able to keep the system reliable. Therefore, based on the failure definition of the railcar system, the system is considered as unreliable when one or more instances of its scenario fail. If the number of failure instances (NFI) of scenario *j* is represented as $NFI_{j}$ and its possible number of active scenario instances as $x_{j}$, one must calculate $P(NFI_{j}=1)$ out of $x_{j}$ active scenario instances. Based on the binomial distribution definition [44], $NFI_{j}$ is a random variable that follows a binomial distribution. As the binomial distribution gives the probability of the possible number of successes (the successes here will refer to failures) in *N* trials (refers to the number of active instances of the scenario) for independent events. Each has an occurrence probability *p* (mapped to the failure probability of scenarios). Using the probability of failure of the scenario *j* $\left(fSc_{j}\right)$ that already has been calculated through Equation (4) and the reliability of scenario *j*$\left(RSc_{j}\right)$ calculated by Equation (5) as input to the formula of the binomial distribution defined in Equation (8), the $P(NFI_{j}=1)$ can easily be calculated.

(8) $$P\left(NFI_{j}\right)=\frac{x_{j}^{!}}{NFI_{j}!\left(x_{j}-NFI_{j}\right)!}f_{Sc_{j}}^{NFI_{j}}{\left(R_{Sc_{j}}\right)}^{x_{j}-NFI_{j}}$$

In the railcar system example, based on Equation (8), the *P*($NFI_{1}=1$) for scenario three is calculated as follows:

$$P\left(NFI_{3}=1\right)=\frac{3}{1!(3-1)!}{\left(0.047004\right)}^{1}{\left(0.952996\right)}^{3-1}=0.12806$$

Similarly, $P(NFI_{1}=1)=0.156498$, and $P(NFI_{2}=1)=0.185142$. Since any scenario instance failure directly leads to the whole system failing, the reliability of a combination, $R_{comb}\left(x_{1},x_{2},\ldots ,x_{S}\right)$ can be defined as:

(9) $$R_{comb}\left(x_{1},x_{2},\ldots ,x_{S}\right)=1-\sum_{\forall x_{S}\neq 0}P\left(NFI_{j}\right)$$

*S* denotes the total number of different scenarios in the system. To complete the prediction of the railcar system, the results that have been calculated using Equation (8) are combined according to Equation (9) and are shown as follows:

$$R_{comb}\left(x_{1}=1,x_{2}=0,x_{3}=2\right)=1-\left(0.156498+0.128067\right)=0.715435$$

This combination above was selected here as an example, and already it has been interpreted in the discussion of Table 3’s values. Thus, similarly, the $R_{comb}$ for all the possible combinations depicted in Table 3 can be calculated.

#### 3.3.4. Calculating the Reliability of the System

As a final step, the concurrent system’s reliability is calculated by combining the results obtained from the previous steps. The reliability of the sequential system is calculated as a weighted mean of scenario reliabilities, and the reliability of the concurrent system is calculated as a weighted mean of the scenario combinations’ reliabilities. The weights represent the occurrence probabilities of the combinations shown in Table 3. Therefore, if the system consists of *k* possible combinations, that system’s reliability can be calculated using Equation (10).

(10) $$R_{sys}=\frac{\sum_{k=1}^{K}W_{k}R_{comb_{j}}}{\sum_{k=1}^{K}W_{k}}$$

$W_{k}$ refers to the occurrence probability of combination *k*, and *K* is the total number of the combinations.

In the case of the railcar system, according to Equation (10) and the data shown in Table 4, the system’s reliability is 0.672685. Note that the maximum number of allowed active scenario instances at a time is three. Therefore, this value was calculated for the worst-case complexity when three active scenarios are running in the system simultaneously. This reliability value can be considered a snapshot of the system reliability because the reliability is calculated when the active scenarios are precisely equal to three (n=3). In order to complete the image of the system reliability, using a similar method to that used for n=3, the reliability can be calculated for distinct cases regarding *n*s value (by taking n≤3), not only in one case. Additionally, this can provide a range of system reliability values that cover the points n=0, n=1, n=2 and n=3.

## 4. Evaluation

In this section, the proposed model is evaluated by comparing its performance with current models. The evaluation explores the computational cost reduction regarding the space needed to deal with the generated state model. The proposed model was compared with a hierarchical [36] model and a composition model [37]. These two models [35,36] were selected because they are similar to our proposed model by providing solutions to the scalability problem and by considering the concurrency, as discussed previously in Section 2. Moreover, the selected models use UML-like notation to convert system specifications to MM. This can be considered an essential factor for a model to be promising. As discussed in Section 2, a number of the models shown in Table 1 use special tools that are not available to download or as commercial products to be obtained for comparison. Therefore, they were neglected.

The hierarchical model was introduced by [36] and also used another way in [19]. In the hierarchical model, the system’s reliability is calculated through the dividing of each scenario into small parts depending on a hierarchical organization. The composition model presented by [37] was used to generate and calculate the reliability by applying a parallel composition to component models. Both models transform system specifications into state machines to be input for calculating system reliability. In the hierarchical or composition model, there are two factors affecting the state space, and therefore the corresponding computational effort. These factors are the maximum number of states (Ss) within an individual state machine and the number of state machines (SMs). In the hierarchical model, Ss are small and SMs are big, and in the compositional model, the situation is inverse. Therefore, our goal in the comparison was to demonstrate that our proposed model can reduce (1) MSs compared to the hierarchical model and (2) Ss compared to the compositional model. Ss and SMs are the main factors that can hinder the model’s scalability. On the other hand, there is a reverse relation between the Ss and SMs (the main elements of the state space explosion problem) and the model scalability. Therefore, decrements in Ss and SMs will enhance the scalability. In the evaluation, the proposed model was applied to various case studies, with different settings regarding the numbers of components and scenarios. Only representative results obtained from railcar system case study are presented in this section.

### The Results

**Figure 6:**
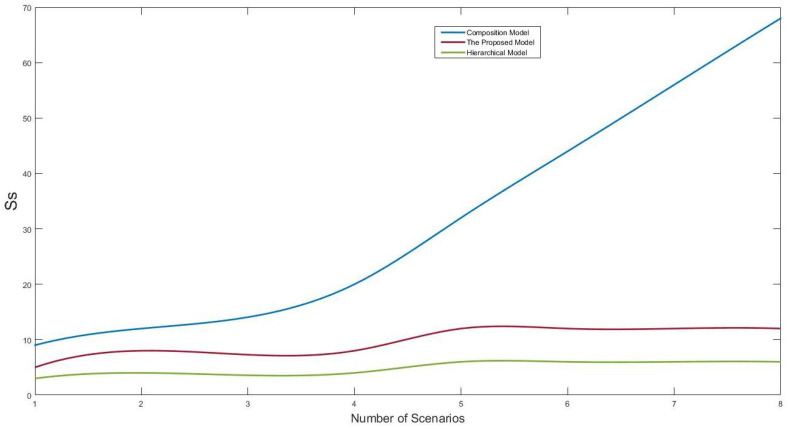
The Ss are generated by the proposed model and the comparison models.

**Figure 7:**
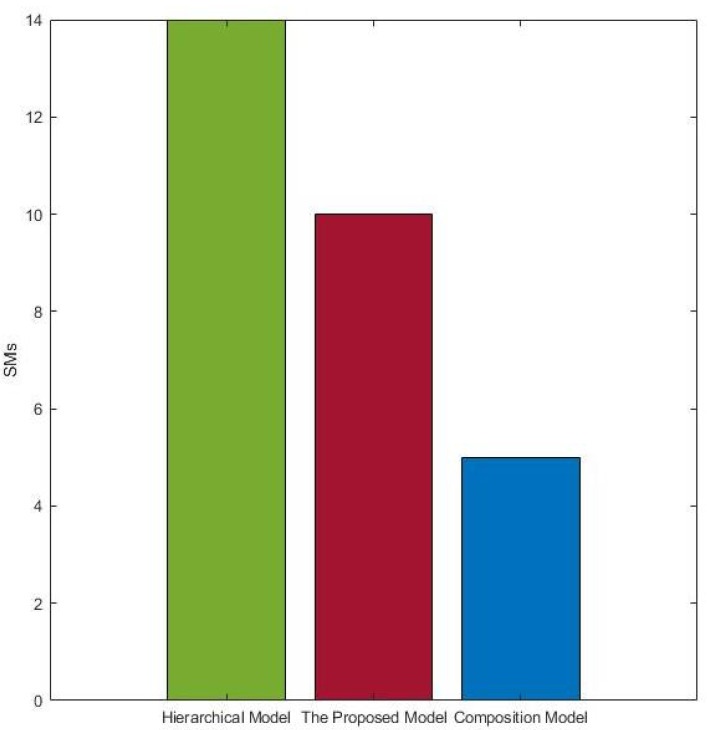
SMs generated by the proposed models and the others.

The results are based on the counting of Ss and SMs after modeling the case study using our proposed model and the comparison models. The proposed model and hierarchy model produced significantly smaller Ss than the composition model. This happened due to the division of the scenario into small units based on the component instances within the individual scenarios, without any regard for the generated state machines. Figure 6 depicts the computational costs saved in practice from the perspective of Ss. The results were obtained based on the scenarios of the railcar system. As shown in Figure 6, the Ss generated by the proposed model and hierarchical model were much less and grew significantly slower than those generated by the composition model. Exploring the SMs generated by each model is necessary to judge the efficiency of computation cost saving. Therefore, Figure 7 shows the SMs generated by each model.

In Figure 6, the hierarchical model demonstrates the best performance regarding Ss. In contrast, in Figure 7, the hierarchical model shows the worst performed regarding SMs. This result clearly confirms that the proposed model performs better than the selected models. Both factors that increase the computational cost, Ss and SMs, have been taken into account. One of the main reasons for reducing SMs is using *s-TS*, which makes compact and concise scenarios. The hierarchical and composition models used the basic form of SMs scenario language, which has no similar properties to *s-TS*. Table 5 summarizes the results shown in Figure 6 and Figure 7.

Table 5 is a tabular representation of Figure 6 and Figure 7. It shows lows and highs based on the Ss and SMs. As shown previously in Figure 6, the composition model produced around 70 Ss, the highest among the models, and it produced less than 5 SMs, the lowest number in Figure 7. Thus, it is marked as high and low for those metrics, respectively, in the table. Based on the performances shown in the figures, the hierarchical model moves from less than 5 Ss to more than 30 SMs, and the proposed model produced between 10 and 15 of the *Ss* and *SMs*. Therefore, the hierarchy is marked low and high for the aforementioned metrics, and the proposed model is marked as medium and medium. The evaluation results clearly shows the effectiveness of the proposed model in the decrementation of Ss and SMs compared to similar models. Therefore, based on the reverse relation between Ss and SMs with the scalability, decrementing their values will enhance scalability.

## 5. Conclusions

The proposed model introduced a new system-level scenario description language named *s-TS* and a modified reliability calculation formula. The *s-TS* helps in describing system scenarios in concisely and compactly manner to reduce the number of system scenarios. In the reliability calculation, the scenario is truncated to be at the level of components and operations rather than the system level for more precision and to avoid state explosion problems. This way of modeling and calculation mitigates computational complexity which is restricting the model’s scalability. The main factors that can lead to controlling complexity are (1) a reduction of the number of state machines (SMs) and (2) a reduction of the states (*Ss*) within the state machine itself. The proposed model achieved a medium number of SMs and a medium number of the Ss compared to the current models. One of the comparison models produced high SMs, and the other model produced a large number of Ss.

A multinomial statistical distribution was adopted in the proposed model for scenario combinations to calculate system reliability. This adaptation allows us to represent the nature of concurrent software applications wherein the number of scenarios running simultaneously is unknown. Therefore, software architects can increase or decrease the number of running scenarios in reliability calculations to know under what load the system will be reliable. In our future work, we plan to perform user studies to evaluate the usability and practical utility of the introduced scenario description language, *s-TS*, through the development of open-source tools. *s-TS* can be used independently as a modeling tool. Furthermore, we plan to add the proposed reliability prediction model as part of the tool to make the model available to public users and enrich the evaluation results.

## Figures and Tables

**Table 1:** Comparison of design-time reliability prediction models.

Model	Year	Behavior Model Notations	Reliability Calculation Model	Concurrency	Operation-Level	Scalability
Singh et al. [36]	2001	UCD + SDs	scenarios failure formula + bayesian	×	(✓)	(✓)
Reussner et al. [25]	2003	RADL	DTMC	×	✓	×
Yacoub et al. [30]	2004	MSCs	PDG + algorithm	×	×	×
Rodrigues et al. [35]	2005	MSCs	DTMC	×	(✓)	(✓)
Roshandel et al. [27]	2007	MM	DTMC + bayesian	×	(✓)	×
Hsu and Huang [31]	2011	MM	path failure formulas + semi truncation strategy	(✓)	(✓)	(✓)
Brosch et al. [7]	2011	PCM	DTMC + algorithms + simulation	(✓)	✓	×
Benes et al. [28]	2012	CAWPT	DTMC + PLT-logic based model	×	✓	×
Tyagi and Sharma [32]	2012	Text-based	fuzzy-logic-based model	(✓)	×	×
Cheung et al. [16]	2012	SDs + MM	CTMC + Truncation strategy	(✓)	(✓)	(✓)
El Kharboutly and Gokhale [33]	2014	MM	CTMC + scenarios compressing	×	(✓)	(✓)
Hou et al. [19]	2018	UCD + MM	PDG + algorithm	×	×	×
Tarinejad et al. [13]	2021	MM	DTMC	×	×	×
Our proposed model	2022	*s-TS* + FSMS	modified scenarios failure formula + multinomial distribution	✓	✓	✓

**Table 2:** Operations’ failure information for the password verification scenario.

Operation	Failure Probability
insertCard	0.002
enterPass	0.01
check	0.004
CheckResult	0.003
DIS_option	0.003
PassIncorrect	0.003

**Table 3:** The combination probabilities $p(x_{1},x_{2},x_{3})$ of the scenarios in the railcar system under the assumption $x_{n}\leq 3$.

The Number of Active Scenarios		$x_{3}$			
$x_{1}$	$x_{2}$	0	1	2	3
0	0	1.000	0.200	0.040	0.008
0	1	0.500	0.200	0.060	0
0	2	0.250	0.150	0	0
0	3	0.125	0	0	0
1	0	0.300	0.120	0.036	0
1	1	0.300	0.180	0	0
1	2	0.225	0	0	0
1	3	0	0	0	0
2	0	0.090	0.054	0	0
2	1	0.135	0	0	0
2	2	0	0	0	0
2	3	0	0	0	0
3	0	0.027	0	0	0
3	1	0	0	0	0
3	2	0	0	0	0
3	3	0	0	0	0

**Table 4:** The combination probabilities of the scenarios in the railcar under the assumption that there are precisely three scenario instances running at the same time $\left(x_{n}=3\right)$.

The Number of Active Scenario Instances		$x_{3}$			
$x_{1}$	$x_{2}$	0	1	2	3
0	0	0	0	0	0.008
0	1	0	0	0.060	0
0	2	0	0.150	0	0
0	3	0.125			
1	0			0.036	
1	1		0.180		
1	2	0.225			
2	0	0	0.054	0	0
2	1	0.135	0	0	0
3	0	0.027	0	0	0

**Table 5:** The summary of the comparison results.

Factors Lead to High Computational Cost	Ss	SMs
Hierarchical model	Low	High
Composition model	High	Low
Proposed model	Medium	Medium

**Figure A1:**
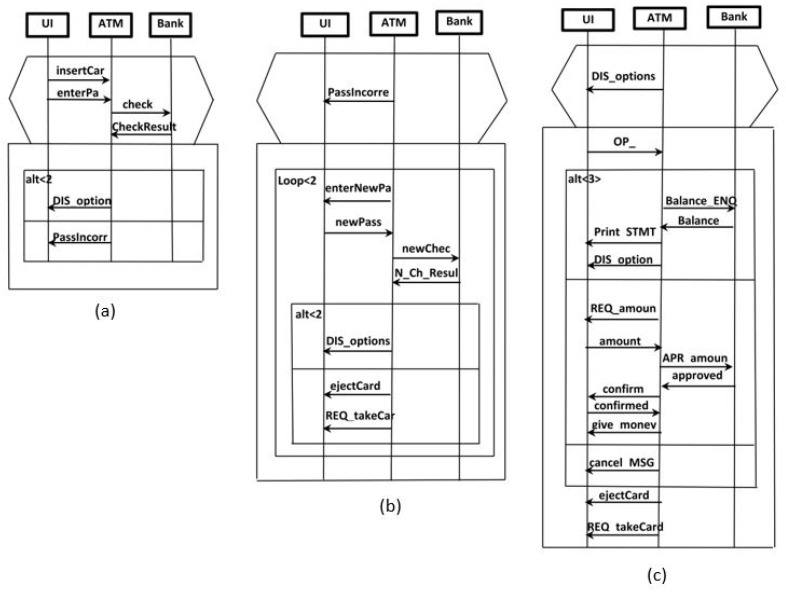
System specifications of ATM case study in form of *s-TS*. (**a**) Scenario of card insertion (scenario 1). (**b**) Scenario of incorrect password (scenario 2). (**c**) Scenario of display options (scenario 3).

**Figure A2:**
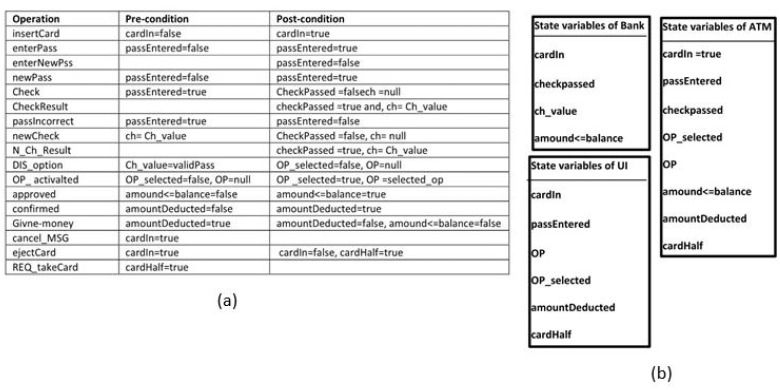
System constraints and state variables that were used for annotation of *s-TS* scenarios. (**a**) System constraints table of ATM case study. (**b**) State variables of the components of ATM case study.

**Figure A3:**
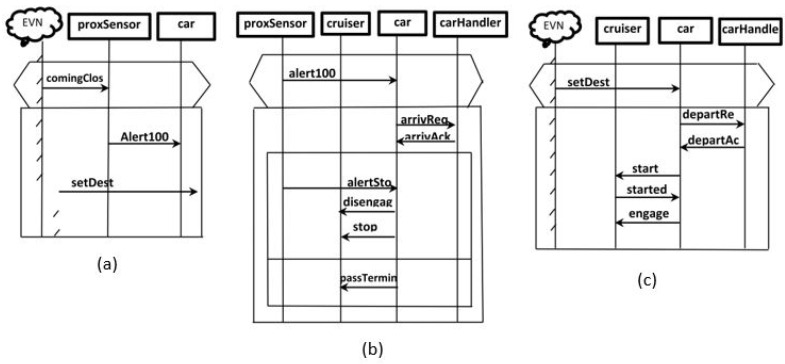
System Specifications of Railcar Case Study in the Form of *s-TS*. (**a**) Scenario of passenger in terminal (scenario 1). (**b**) Scenario of car approaching terminal (scenario 2). (**c**) Scenario of car departing terminal (scenario 3).

**Figure A4:**
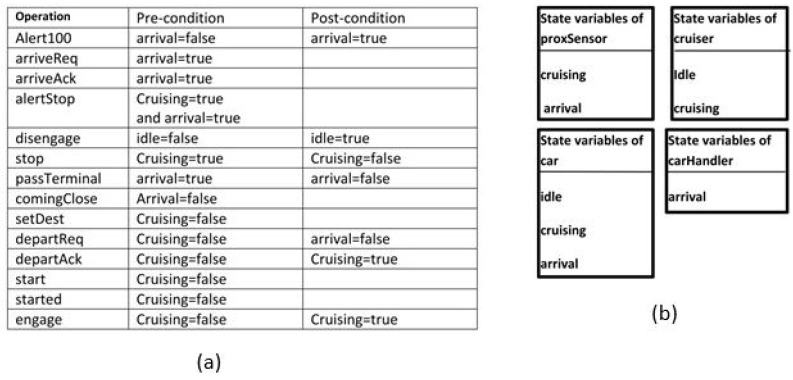
System Constraints Table and State Variables of the Components of the Railcar Scenario. (**a**) System constraints table of of railcar. (**b**) State variables of the components of railcar.

## Data Availability

Not applicable.

## Appendix A

Appendix A contains the artifacts related to the ATM case study that were used as inputs for the proposed model.

## Appendix B

Appendix B contains the artifacts related to the railcar case study used as inputs for the proposed model.

## References

[1] Immonen A., Niemelä E. (2008). Survey of reliability and availability prediction methods from the viewpoint of software architecture. Softw. Syst. Model..

[2] Musa J.D., Iannino A., Okumoto K. (1987). Software Reliability: Measurement, Prediction, Application.

[3] Roy B., Graham T.N. (2008). Methods for evaluating software architecture: A survey. Sch. Comput. TR.

[4] Wohlin C., Runeson P. A method proposal for early software reliability estimation. Proceedings of the 3rd International Symposium on Software Reliability Engineering (ISSRE).

[5] Cukic B. (2005). The virtues of assessing software reliability early. IEEE Softw..

[6] Cheung L., Roshandel R., Medvidovic N., Golubchik L. Early prediction of software component reliability. Proceedings of the 30th International Conference on Software Engineering.

[7] Brosch F., Koziolek H., Buhnova B., Reussner R. (2011). Architecture-based reliability prediction with the palladio component model. IEEE Trans. Softw. Eng..

[8] Sibay G.E., Braberman V., Uchitel S., Kramer J. (2012). Synthesizing modal transition systems from triggered scenarios. IEEE Trans. Softw. Eng..

[9] Krka I., Medvidovic N. Component-aware triggered scenarios. Proceedings of the 2014 IEEE/IFIP Conference on Software Architecture.

[10] Whittle J., Jayaraman P.K. (2010). Synthesizing hierarchical state machines from expressive scenario descriptions. ACM Trans. Softw. Eng. Methodol..

[11] Torre D., Labiche Y., Genero M., Baldassarre M.T., Elaasar M. UML diagram synthesis techniques: A systematic mapping study. Proceedings of the 10th International Workshop on Modelling in Software Engineering.

[12] Ali A., Jawawi D., Isa M.A. (2015). Scalable scenario specifications to synthesize component-centric behaviour models. Int. J. Softw. Eng. Appl..

[13] Tarinejad A., Izadkhah H., Ardakani M.M., Mirzaie K. (2021). Metrics for assessing reliability of self-healing software systems. Comput. Electr. Eng..

[14] Wang W.L., Pan D., Chen M.H. (2006). Architecture-based software reliability modeling. J. Syst. Softw..

[15] Chen L., Huang L., Li C., Wu X. (2017). Incorporating architectural modelling with state-based reliability evaluation. Int. J. Hoc Ubiquitous Comput..

[16] Cheung L., Krka I., Golubchik L., Medvidovic N. Architecture-level reliability prediction of concurrent systems. Proceedings of the 3rd ACM/SPEC International Conference on Performance Engineering.

[17] Cooray D., Kouroshfar E., Malek S., Roshandel R. (2013). Proactive self-adaptation for improving the reliability of mission-critical, embedded, and mobile software. IEEE Trans. Softw. Eng..

[18] Ali A., NA Jawawi D., Adham Isa M., Imran Babar M. (2016). Technique for early reliability prediction of software components using behaviour models. PLoS ONE.

[19] Hou C., Wang J., Chen C. (2018). Using hierarchical scenarios to predict the reliability of component-based software. IEICE Trans. Inf. Syst..

[20] Krka I., Edwards G., Cheung L., Golubchik L., Medvidovic N. (2009). A comprehensive exploration of challenges in architecture-based reliability estimation. Architecting Dependable Systems VI.

[21] Mosimann J.E. (1962). On the compound multinomial distribution, the multivariate *β*-distribution, and correlations among proportions. Biometrika.

[22] Harel D., Gery E. Executable object modeling with statecharts. Proceedings of the IEEE 18th International Conference on Software Engineering.

[23] Al-Fedaghi S. (2021). Diagrammatic Formalism for Complex Systems: More than One Way to Eventize a Railcar System. arXiv.

[24] Harel D., Marelly R., Marron A., Szekely S. (2020). Integrating Inter-Object Scenarios with Intra-object Statecharts for Developing Reactive Systems. IEEE Des. Test.

[25] Reussner R.H., Schmidt H.W., Poernomo I.H. (2003). Reliability prediction for component-based software architectures. J. Syst. Softw..

[26] Goševa-Popstojanova K., Trivedi K.S. (2001). Architecture-based approach to reliability assessment of software systems. Perform. Eval..

[27] Roshandel R., Medvidovic N., Golubchik L. A Bayesian model for predicting reliability of software systems at the architectural level. Proceedings of the International Conference on the Quality of Software Architectures.

[28] Benes N., Buhnova B., Cerna I., Oslejsek R. Reliability analysis in component-based development via probabilistic model checking. Proceedings of the 15th ACM SIGSOFT symposium on Component Based Software Engineering.

[29] ChauPattnaik S., Ray M., Nayak M.M. (2021). Component based reliability prediction. Int. J. Syst. Assur. Eng. Manag..

[30] Yacoub S., Cukic B., Ammar H.H. (2004). A scenario-based reliability analysis approach for component-based software. IEEE Trans. Reliab..

[31] Hsu C.J., Huang C.Y. (2011). An adaptive reliability analysis using path testing for complex component-based software systems. IEEE Trans. Reliab..

[32] Tyagi K., Sharma A. (2012). A rule-based approach for estimating the reliability of component-based systems. Adv. Eng. Softw..

[33] El Kharboutly R., Gokhale S.S. (2014). Efficient reliability analysis of concurrent software applications considering software architecture. Int. J. Softw. Eng. Knowl. Eng..

[34] Babeker A.A.M.E. (2015). Quality Measurement Model for Composite Service-oriented Design. Ph.D. Thesis.

[35] Aziz M.W., Radziah M., Jawawi D. (2013). Service-oriented Analysis and Design Approach for Distributed Embedded Real-time Systems. Ph.D. Thesis.

[36] Cheung L., Golubchik L., Medvidovic N. SHARP: A scalable approach to architecture-level reliability prediction of concurrent systems. Proceedings of the 2010 ICSE Workshop on Quantitative Stochastic Models in the Verification and Design of Software Systems.

[37] Rodrigues G., Rosenblum D., Uchitel S. (2005). Using scenarios to predict the reliability of concurrent component-based software systems. International Conference on Fundamental Approaches to Software Engineering.

[38] Roshandel R., Schmerl B., Medvidovic N., Garlan D., Zhang D. Understanding tradeoffs among different architectural modeling approaches. Proceedings of the Fourth Working IEEE/IFIP Conference on Software Architecture (WICSA 2004).

[39] Singh H., Cortellessa V., Cukic B., Gunel E., Bharadwaj V. A bayesian approach to reliability prediction and assessment of component based systems. Proceedings of the 12th International Symposium on Software Reliability Engineering.

[40] Goseva-Popstojanova K., Hassan A., Guedem A., Abdelmoez W., Nassar D.E.M., Ammar H., Mili A. (2003). Architectural-level risk analysis using UML. IEEE Trans. Softw. Eng..

[41] Sadi M.S., Myers D., Sanchez C.O., Jurjens J. (2010). Component criticality analysis to minimizing soft errors risk. Comput. Syst. Sci. Eng..

[42] Johnson N.L. (1997). Discrete Multivariate Distributions.

[43] Zelterman D. (2014). Multinomial Distribution: Overview. Wiley StatsRef: Statistics Reference Online.

[44] Lane D. Hyperstat Online: An Introductory Statistics Textbook and Online Tutorial for Help in Statistic. https://davidmlane.com/hyperstat/.

